# Cystic Schwannoma of the Lumbar Spine Presenting as a Solid Lesion During Surgery: Radiologic-Pathologic Correlation

**DOI:** 10.7759/cureus.107029

**Published:** 2026-04-14

**Authors:** Mamadou Bata Dianka, Idania Cruzata Matos, Yelka Matos Furones, Elizabeth Blanco Espinosa, Jose I Gamboa Arisso, Luis F Gonzalez Vazquez

**Affiliations:** 1 Neurosurgery, Hospital Général Peltier, Djibouti, DJI; 2 General Medicine, HCA Healthcare, Nevada, USA; 3 Neurology, North Georgia Clinical Research, Alcanza Clinical Research, Woodstock, USA; 4 General Surgery, Neurosurgery, Hospital Arnaldo Milian, Santa Clara, CUB; 5 General Practice, Ceda Orthopedic Group, Miami, USA; 6 General Medicine, Jimmy Hirtzel Hospital, Miami, USA; 7 Anesthesiology, Geisinger Medical Center, Danville, USA

**Keywords:** cystic schwannoma, cystic spinal lesion, intradural extramedullary tumor, laminectomy, lumbar spine tumor, magnetic resonance imaging, nerve sheath tumor, radiculopathy, radiologic-pathologic correlation, spinal schwannoma

## Abstract

Spinal schwannomas are common benign intradural extramedullary tumors that typically present as solid lesions on imaging. However, cystic degeneration may occur and produce atypical radiological features, complicating preoperative diagnosis and differential considerations. We report the case of a 46-year-old woman presenting with a three-month history of left-sided lumbosciatica and intermittent paresthesia without neurological deficit. Magnetic resonance imaging revealed a well-circumscribed intradural cystic lesion anterior to the L4 vertebral body with peripheral contrast enhancement, suggestive of a cystic schwannoma. The patient underwent L3-L4 laminectomy and microsurgical resection. Intraoperatively, the lesion was found to be predominantly solid despite its cystic radiological appearance, highlighting a radiologic-intraoperative discordance. Histopathological analysis confirmed the diagnosis of cystic schwannoma, demonstrating spindle-shaped Schwann cells, myxoid stroma, and focal degenerative changes. The postoperative course was uneventful, with complete symptom resolution and no evidence of residual tumor on follow-up imaging. Cystic schwannomas represent a diagnostic challenge due not only to their heterogeneous imaging characteristics but also to potential discrepancies between imaging and intraoperative findings. The correlation between imaging, surgical findings, and histopathology is essential for accurate diagnosis. This case highlights the importance of considering schwannoma in the differential diagnosis of intradural cystic spinal lesions, even when imaging suggests a purely cystic mass. Complete microsurgical resection remains the treatment of choice and is associated with excellent clinical outcomes.

## Introduction

Spinal schwannomas are among the most common benign tumors arising within the spinal canal and originate from Schwann cells of the peripheral nerve sheath. Epidemiological analyses of intradural spinal tumors confirm that schwannomas constitute a substantial proportion of benign intradural lesions and frequently arise from dorsal nerve roots. These tumors predominantly affect adults, particularly individuals in middle age, and typically demonstrate slow growth patterns that may allow them to remain clinically silent for prolonged periods before producing symptoms related to neural compression [1].

Intradural extramedullary tumors represent a major category of spinal neoplasms located within the dural sac but outside the spinal cord parenchyma. Among these, schwannomas and meningiomas are the most frequently encountered entities. Accurate identification of these tumors prior to surgical intervention is essential, as treatment planning and prognostic considerations depend heavily on tumor type and anatomical relationships. Magnetic resonance imaging (MRI) plays a central role in distinguishing these tumors from other intradural pathologies and in guiding clinical management [2,3].

MRI with contrast enhancement remains the imaging modality of choice for the evaluation of spinal tumors because of its superior soft tissue contrast and multiplanar capabilities. MRI enables detailed visualization of tumor morphology, signal characteristics, and relationships with adjacent neural structures. These imaging features are particularly useful in differentiating schwannomas from other intradural extramedullary tumors and in identifying atypical variants [3].

Cystic lesions of the spinal canal present a significant diagnostic challenge because they encompass a wide spectrum of pathological entities, including arachnoid cysts, synovial cysts, and tumors with cystic degeneration. Careful radiological evaluation is required to distinguish between these conditions. Although schwannomas are typically described as solid tumors, they may occasionally demonstrate cystic components, which can obscure their classic imaging appearance and complicate the differential diagnosis [4].

Recent studies have shown that cystic degeneration may occur in a subset of spinal schwannomas. Systematic reviews of intradural cystic schwannomas have demonstrated that these tumors can exhibit heterogeneous imaging patterns and occasionally mimic other cystic spinal lesions, making accurate preoperative diagnosis challenging [5].

Rare cases of schwannomas presenting as predominantly or entirely cystic lesions have also been reported, particularly in the lumbar spine, further highlighting the importance of including schwannoma in the differential diagnosis of intradural cystic masses [6]. Although most schwannomas occur in the intradural extramedullary compartment, rare variants have been reported in unusual anatomical locations, including intramedullary regions or tumors with both intra- and extraspinal extension. However, these aspects are less relevant in cases where the primary diagnostic challenge arises from atypical imaging characteristics [7].

Surgical resection remains the treatment of choice for spinal schwannomas. Several studies have demonstrated that complete tumor removal is associated with favorable neurological outcomes and long-term disease control. The benign nature and slow growth of these tumors contribute to excellent postoperative prognosis when early diagnosis and appropriate surgical management are achieved [8]. Various surgical approaches have been described depending on tumor size, location, and relationship with surrounding neural structures. Laminectomy remains one of the most used techniques because it provides adequate exposure of the spinal canal and facilitates safe tumor removal while minimizing neurological morbidity [9].

In some cases, particularly when tumors are present with large extraspinal components or extensive cystic degeneration, more complex surgical strategies may be required to achieve complete resection [10]. An additional and clinically relevant challenge is the potential discordance between radiological and intraoperative findings. Lesions that appear predominantly cystic on MRI may be found to be solid during surgery, which can lead to diagnostic uncertainty and influence surgical planning and intraoperative decision-making.

In this report, we present the case of a lumbar intradural cystic schwannoma with radiologic-intraoperative discordance. The case highlights the diagnostic variability of cystic spinal schwannomas and underscores the importance of correlating imaging, surgical, and histopathological features for accurate diagnosis and management.

## Case presentation

History and clinical findings

A 46-year-old woman presented with a three-month history of progressive left-sided lumbosciatica associated with intermittent paresthesia. The pain radiated along the posterior aspect of the left lower limb in a sciatic distribution and was exacerbated by prolonged sitting and physical activity, partially relieved with rest and analgesics. She denied bowel or bladder dysfunction, saddle anesthesia, gait instability, or constitutional symptoms, such as fever, weight loss, or night sweats.

Her past medical history was unremarkable, with no prior spinal trauma, surgery, or known malignancy.

Neurological examination

On examination, motor strength was preserved in all lower extremity muscle groups (Medical Research Council grade 5/5) [11]. Sensory testing to light touch and pinprick was intact bilaterally. Deep tendon reflexes were symmetric, and no pathological reflexes were elicited. Both the Lasègue (straight leg raise) and Bragard tests were negative bilaterally. There were no signs of myelopathy, radiculopathy, or focal neurological deficit at the time of evaluation. Despite the absence of objective neurological deficits and negative provocative tests, the patient's symptoms were clinically consistent with radicular irritation, likely related to mild neural displacement caused by the intradural lesion observed on imaging.

Laboratory findings

Laboratory investigations were performed as part of the initial evaluation (Table 1).

**Table 1:** Summary of laboratory findings on admission Laboratory values were within normal limits and did not contribute to the diagnostic or therapeutic decision-making in this case.

Parameter	Result	Reference Range	Interpretation
White blood cell count	5.97 × 10³/µL	4.0-11.0 × 10³/µL	Normal
Neutrophils (%)	38.10%	40.0-70.0%	Mildly decreased
Eosinophils (%)	9.00%	1.0-6.0%	Mildly elevated
Red blood cell count	4.21 × 10⁶/µL	4.0-5.2 × 10⁶/µL	Normal
Hemoglobin	12.4 g/dL	12.0-16.0 g/dL	Normal
Platelet count	285 × 10³/µL	150-450 × 10³/µL	Normal

Neuroimaging findings

**Figure 1:**
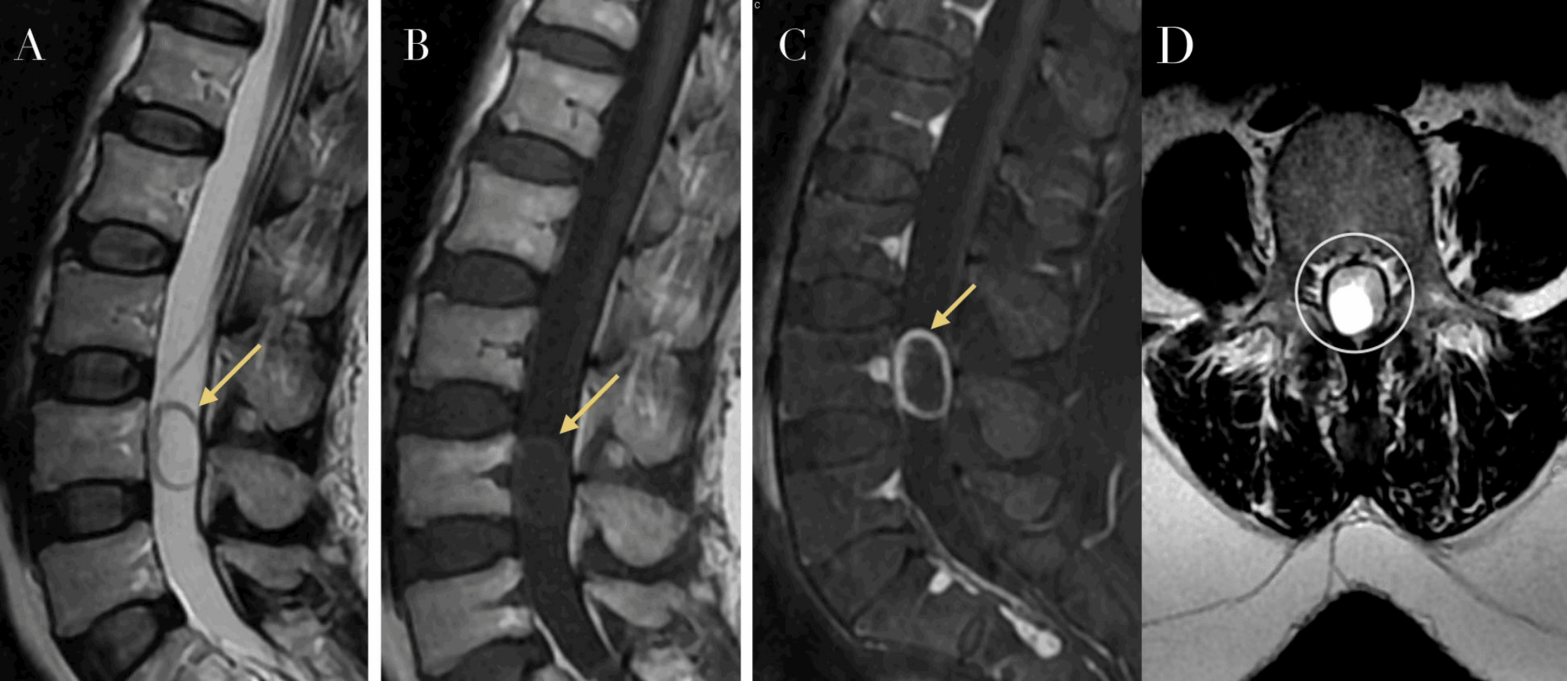
Lumbar spine MRI showing a cystic intradural lesion (A) T2-weighted image showing a hyperintense intradural lesion anterior to L4 (yellow arrow). (B) T1-weighted image demonstrating corresponding hypointensity (yellow arrow). (C) Post-gadolinium T1 image with peripheral rim enhancement (yellow arrow). (D) Axial T2 MRI showing a hyperintense intradural lesion with mild mass effect, consistent with a cystic tumor (yellow arrow).

MRI of the lumbar spine with gadolinium contrast, performed on a 1.5-Tesla system, demonstrated a well-circumscribed oblong intradural lesion located anterior to the L4 vertebral body. The lesion measured approximately 25 × 19 × 14 mm. It appeared hypointense on T1-weighted images and hyperintense on T2-weighted sequences, with peripheral contrast enhancement following gadolinium administration. The lesion exhibited a smooth, moderately thick wall and caused mild displacement of adjacent neural elements without clear evidence of nerve root invasion. Axial T2-weighted images showed a well-defined hyperintense intradural lesion centrally located within the spinal canal, causing mild displacement of adjacent neural structures without evidence of invasion, consistent with a cystic lesion (Figure 1).

These radiological features were suggestive of an intradural cystic tumor, with cystic schwannoma considered the leading differential diagnosis. Other differential diagnoses included arachnoid cyst, neurenteric cyst, and cystic meningioma; however, the presence of peripheral contrast enhancement and a well-defined capsule favored a neoplastic etiology, particularly schwannoma, over non-enhancing cystic lesions, such as arachnoid cysts.

Additionally, a moderate disc protrusion at L5-S1 was observed without significant canal stenosis or nerve root compression. This finding was considered incidental and unlikely to account for the patient's symptoms.

Surgical management

The patient underwent surgical resection through a standard L3-L4 laminectomy under general anesthesia. After durotomy, a well‑defined, firm intradural lesion was identified, located dorsal to the remaining cauda equina roots and producing focal thickening of a single lumbar nerve root. Based on the clinical presentation and MRI findings, the involved root most likely corresponded to L4, although precise identification was limited by the absence of microsurgical adjuncts and the natural convergence of the cauda equina roots at the L4-L5 level. The affected root demonstrated focal enlargement consistent with a schwannoma, without dural attachment or dural tail. Despite preoperative imaging suggesting a cystic component, the lesion was entirely solid, with no cystic cavity, fluid collection, or cyst collapse encountered intraoperatively. The tumor was surrounded by a firm, well‑defined capsule typical of schwannomas. Meticulous dissection allowed careful mobilization of the mass while preserving the involved nerve root, and the lesion was removed completely without complications. Hemostasis was achieved, and the procedure concluded uneventfully.

Histopathological findings

Gross examination revealed a specimen measuring 2.5 × 1.8 cm. Microscopically, the tumor consisted of spindle-shaped Schwann cells arranged in loose fascicles with areas of low cellularity. Rudimentary Verocay bodies were identified. The lesion was partially encapsulated by a thin fibrous capsule containing nerve fibers. The stroma was predominantly myxoid with focal hemorrhage, hyalinization, and ectatic blood vessels with thickened walls. No significant atypia or mitotic activity was observed. Immunohistochemical analysis was not performed, and the diagnosis was established based on characteristic histomorphological features.

**Figure 2:**
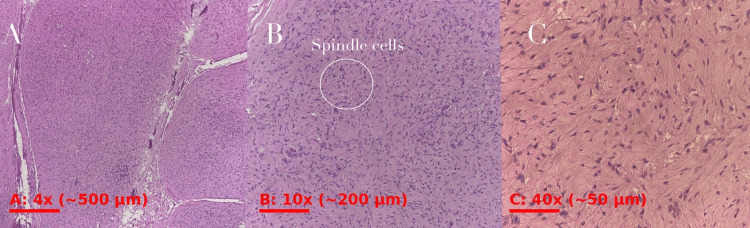
Histopathological findings consistent with cystic schwannoma (A) Low-power view (4×) showing a partially encapsulated lesion with areas of low cellularity. (B) Intermediate magnification (10×) demonstrating spindle-shaped cells arranged in loose fascicles within a myxoid stroma. (C) High-power view (40×) revealing elongated cells with wavy nuclei and no significant atypia or mitotic activity.

These findings were consistent with a cystic schwannoma (Figure 2).

Postoperative course and follow-up

The postoperative course was uneventful. The patient remained hospitalized for 72 hours and received intravenous ceftriaxone, analgesia with paracetamol as needed, and gastric protection according to institutional protocol. She was discharged on postoperative day four with oral analgesics and instructions for outpatient follow-up.

**Figure 3:**
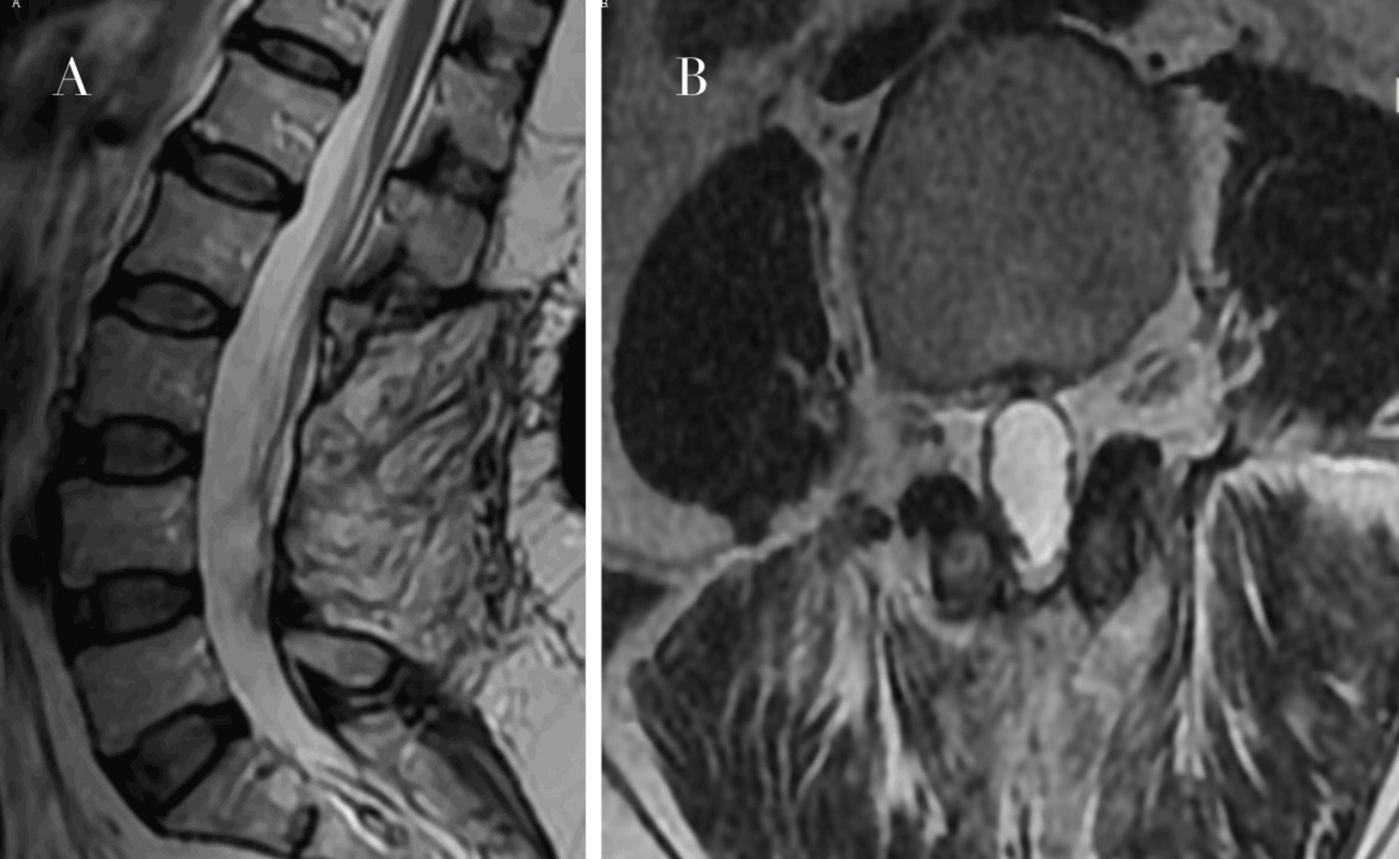
Postoperative lumbar MRI demonstrating complete tumor resection (A) Sagittal view showing restoration of cerebrospinal fluid signal and decompression of the thecal sac without residual mass. (B) Axial view confirming absence of residual tumor and adequate decompression of the spinal canal.

Postoperative MRI confirmed complete resection of the lesion with no evidence of residual tumor (Figure 3).

At one-month follow-up, the patient reported complete resolution of radicular pain and paresthesia. Neurological examination remained normal, with no evidence of recurrence or functional impairment. A longer follow-up period was not available, which represents a limitation of this report.

Ethical approval

In accordance with the policies of the hosting institution, formal ethics committee approval was not required for the publication of this single case report. The patient provided written informed consent, permitting the use and publication of anonymized clinical information and associated images. All appropriate measures were implemented to maintain patient confidentiality and safeguard privacy. No identifying personal information is included in this manuscript.

## Discussion

Cystic degeneration in spinal schwannomas is a recognized but relatively uncommon phenomenon that contributes to the heterogeneous presentation of these tumors. Several biological mechanisms have been proposed to explain this process, including progressive vascular compromise, repeated intratumoral microhemorrhages, and myxoid stromal degeneration. These changes may lead to the formation of fluid-filled cavities within the tumor matrix. Histopathologically, cystic degeneration is often associated with the Antoni B pattern, characterized by loosely arranged Schwann cells and abundant extracellular matrix. These structural alterations may explain the atypical imaging patterns sometimes observed in schwannomas that resemble primary cystic lesions of the spinal canal [3,5].

The relationship between cystic transformation and tumor chronicity has also been emphasized in recent studies [5]. Long-standing schwannomas may undergo progressive internal degeneration when tumor growth exceeds vascular supply. This imbalance may result in focal ischemia, necrosis, and subsequent cyst formation within the tumor. Clinical series suggest that cystic degeneration may therefore represent a later stage in the biological evolution of spinal schwannomas, particularly in lesions that remain undiagnosed for prolonged periods [1].

In the present case, the patient's lumbar schwannoma demonstrated this phenomenon, with MRI suggesting a predominantly cystic lesion, whereas intraoperative findings revealed a mostly solid tumor with microscopic cystic spaces, illustrating this radiologic-intraoperative mismatch.

Radiological evaluation plays a crucial role in the diagnosis of spinal schwannomas, yet cystic variants may pose a diagnostic challenge. MRI may demonstrate features such as peripheral contrast enhancement, heterogeneous signal intensity, and well-circumscribed cystic components. These characteristics can mimic other intradural cystic lesions, including arachnoid cysts, neurenteric cysts, epidermoid tumors, or cystic meningiomas. In this case, subtle imaging features, including peripheral enhancement and location anterior to the L4 vertebral body, suggested a schwannoma over other cystic lesions [4,6].

Another important diagnostic consideration is the discrepancy that may occur between radiological and intraoperative findings. Imaging may suggest a predominantly cystic lesion, while surgical exploration reveals a mostly solid tumor with microscopic cystic spaces. This phenomenon may occur when cystic cavities collapse during surgical manipulation or when small fluid-filled areas are dispersed within a predominantly solid tumor matrix. Such discrepancies highlight the importance of histopathological confirmation for definitive diagnosis [4,5]. Standard L3-L4 laminectomy was chosen in this patient to provide optimal exposure for complete microsurgical resection of the lesion while minimizing neurological risk, as the tumor was well-circumscribed and intradural at the L4 level.

Although spinal schwannomas most commonly arise in the intradural extramedullary compartment, rare intramedullary variants have been reported. These tumors are thought to originate from aberrant Schwann cells associated with perivascular nerve fibers or from developmental migration of neural crest cells. Recognition of these unusual variants further underscores the biological diversity of schwannomas and the importance of histopathological evaluation in confirming the diagnosis [7].

Surgical resection remains the treatment of choice for symptomatic spinal schwannomas. Because these tumors are typically well circumscribed and encapsulated, complete microsurgical removal can usually be achieved while preserving neurological function. One important surgical consideration is the management of the involved nerve root. Since schwannomas often arise from sensory nerve roots and displace rather than infiltrate neural tissue, the tumor capsule can frequently be separated from the root during microsurgical dissection. In selected cases, sacrifice of the involved sensory root may be performed without a significant neurological deficit [8].

In this case, the tumor was carefully dissected from the adjacent nerve root, which was preserved, further highlighting the technical considerations in resecting cystic schwannomas. The surgical approach is primarily determined by tumor size, location, and extension. Conventional laminectomy remains the most widely used technique for intradural extramedullary tumors because it provides adequate exposure of the spinal canal and facilitates safe tumor resection. However, recent studies emphasize the importance of preserving spinal stability when possible. Minimally invasive alternatives were considered but deemed less suitable given the lesion's size and intradural location [9-12].

In cases where schwannomas extend beyond the spinal canal, more complex surgical strategies may be required. Large tumors with paraspinal or extraspinal components may necessitate staged procedures or multidisciplinary surgical approaches involving neurosurgical and thoracic or orthopedic teams. Careful preoperative planning and detailed imaging evaluation are essential to achieve complete resection while minimizing complications [10].

The natural history of spinal nerve sheath tumors further supports early intervention in symptomatic patients. Longitudinal imaging studies have shown that although many schwannomas demonstrate slow growth, a subset may exhibit progressive enlargement over time. Continued tumor growth may lead to increasing neural compression and progressive neurological symptoms, emphasizing the importance of clinical monitoring and timely surgical treatment [13].

Large clinical series and meta-analyses have consistently demonstrated excellent outcomes following gross total resection of spinal schwannomas. Complete tumor removal is associated with significant improvement in radicular symptoms, low surgical morbidity, and minimal recurrence rates. These outcomes are literature-supported rather than derived from this single case [8,14].

Finally, recent studies have identified several factors associated with favorable neurological recovery following surgical resection of intradural tumors. Early surgical intervention, smaller tumor size, and absence of severe preoperative neurological deficits are associated with improved postoperative outcomes. Conversely, delayed treatment in the presence of advanced neurological compromise may increase the risk of persistent deficits. These findings highlight the importance of early diagnosis and timely surgical management [15].

## Conclusions

Cystic schwannomas of the lumbar spine represent an uncommon variant of intradural extramedullary tumors and may present with atypical radiological features that complicate preoperative diagnosis. This case illustrates a radiologic-intraoperative mismatch due to microscopic cystic degeneration within an otherwise solid tumor. Definitive diagnosis relies on histopathological evaluation correlating with radiological and surgical findings. Complete microsurgical resection remains the treatment of choice and is associated with excellent neurological outcomes and low recurrence rates, as supported by the literature. Awareness of these diagnostic and surgical considerations is essential for optimal management of patients with suspected cystic spinal schwannomas.
